# Supplementary material for: Preparation and Activity of Hemostatic and Antibacterial Dressings with Greige Cotton/Zeolite Formularies Having Silver and Ascorbic Acid Finishes
Source: Int J Mol Sci. 2023 Dec 4;24(23):17115. doi: 10.3390/ijms242317115 (PMC10706952; doi:10.3390/ijms242317115)
Supplement: Supplementary file 1 [file ijms-24-17115-s001.zip › ijms-2629260-supplementary.pdf]

Supplementary

Preparation and Activity of Dressings Designed for Prolonged Field Care Use: Hemostatic and Antibacterial Properties of Greige Cotton/ Zeolite Formularies comparing Silver and Ascorbic Acid Finishes

J. Vincent Edwards[1*], Nicolette T. Prevost[1], Doug Hinchliffe[1], Sunghyun Nam[1], SeChin Chang[1], Rebecca Hron[1], Crista Madison[1], Jade Smith[1], Erika J. Martin[2], Chelsie N. Poffenberger[3], Michelle M. Taylor[3] and Kirsty Dixon[3]

1 Southern Regional Research Center, United States Department of Agriculture, New Orleans, LA.,70124; vince.edwards@usda.gov; nicolette.prevost@usda.gov, doug.hinchliffe@usda.gov, sunghyun.nam@usda.gov, sechin.chang@usda.gov, rebecca.hron@usda.gov, crista.madison@usda.gov, jade.smith@usda.gov

2 Department of Internal Medicine, School of Medicine, Virginia Commonwealth University, Richmond Virginia, 23298; erika.martin@vcuhealth.org

3 Department of Surgery, School of Medicine, Virginia Commonwealth University, Richmond Virginia, 23298; poffenbergec@vcu.edu, taylormm5@vcu.edu, and kirsty.dixon@vcuhealth.org

* Correspondence: vince.edwards@usda.gov; Tel.: +1-504-286-4360

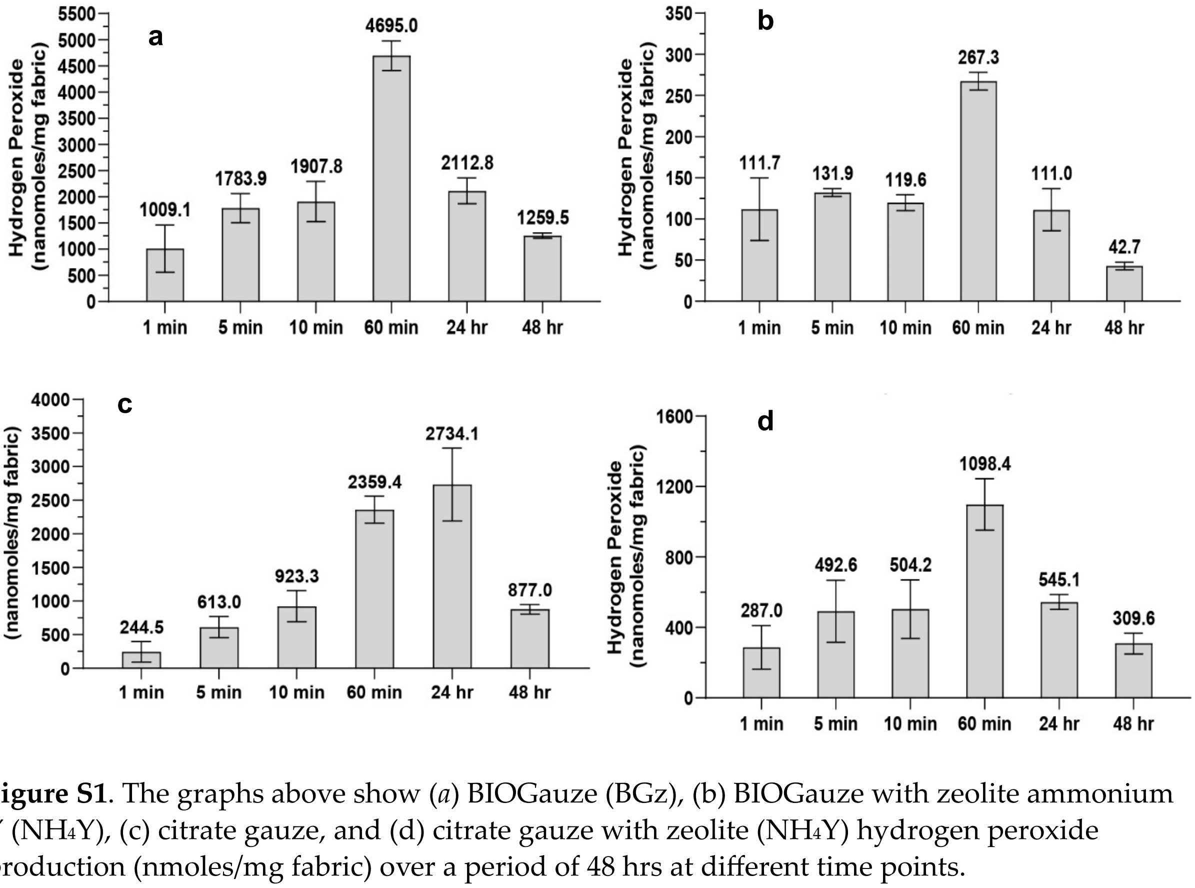

Figure S1. The graphs above show (*a*) BIOGauze (BGz), (b) BIOGauze with zeolite ammonium Y (NH_4Y), (c) citrate gauze, and (d) citrate gauze with zeolite (NH_4Y) hydrogen peroxide production (nmoles/mg fabric) over a period of 48 hrs at different time points.

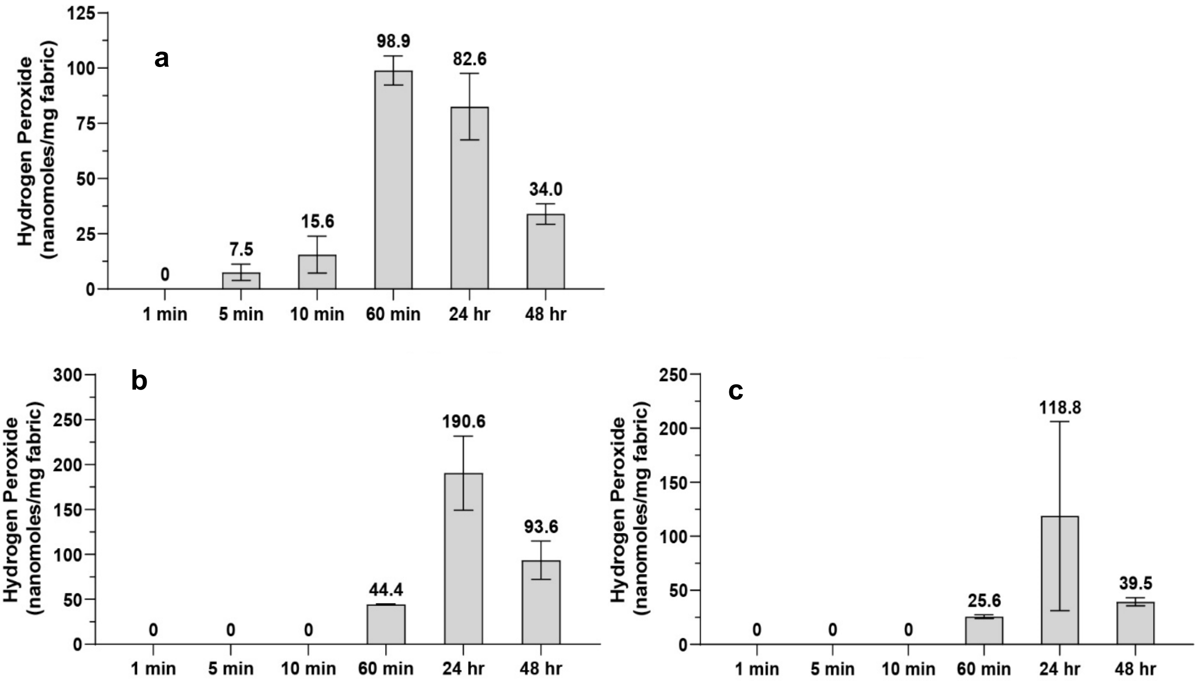

Figure S2. The graphs above show (*a*) TACGauze (TGz), (b) silver TACGauze (AgTGz), and (c) silver TACGauze with zeolite (AgTGz with NH_4Y) hydrogen peroxide production (nmoles/mg fabric) over a period of 48 hrs at different time points.

Table S1. One-way ANOVA of R values shown in Figure 6a as determined by one-way ANOVA with Tukey's post hoc test.

Sample	Mean Diff.	95.00% CI of diff.	Summary	Adjusted p value
Control vs. CG	18.29	14.87 to 21.71	****	<0.0001
Control vs. TGz	10.28	6.856 to 13.69	****	<0.0001
Control vs. NaY	14.29	10.87 to 17.71	****	<0.0001
Control vs. NH_4Y	16.01	12.59 to 19.43	****	<0.0001
Control vs. NH_4Y-$CaCl_2$	16.44	13.02 to 19.86	****	<0.0001
Control vs. NaY (S)	13.78	10.36 to 17.19	****	<0.0001
Control vs. NH_4Y(S)	16.83	13.41 to 20.24	****	<0.0001
CG vs. TGz	-8.017	-11.44 to -4.598	****	<0.0001
CG vs. NaY	-4.000	-7.419 to -0.5809	*	0.0162
CG vs. NH_4Y	-2.283	-5.702 to 1.136	ns	0.3443
CG vs. NH_4Y-$CaCl_2$	-1.850	-5.269 to 1.569	ns	0.5852
CG vs. NaY (S)	-4.517	-7.936 to -1.098	**	0.0059
CG vs. NH_4Y(S)	-1.467	-4.886 to 1.952	ns	0.8044
TGz vs. NaY	4.017	0.5975 to 7.436	*	0.0157
TGz vs. NH_4Y	5.733	2.314 to 9.152	***	0.0006
TGz vs. NH_4Y-$CaCl_2$	6.167	2.748 to 9.586	***	0.0002
TGz vs. NaY (S)	3.500	0.08088 to 6.919	*	0.0429
TGz vs. NH_4Y (S)	6.550	3.131 to 9.969	***	0.0001
NaY vs. NH_4Y	1.717	-1.702 to 5.136	ns	0.6652
NaY vs. NH_4Y-$CaCl_2$	2.150	-1.269 to 5.569	ns	0.4123
NaY vs. NaY (S)	-0.5167	-3.936 to 2.902	ns	0.9993
NaY vs. NH_4Y(S)	2.533	-0.8858 to 5.952	ns	0.2375
NH_4Y vs. NH_4Y-$CaCl_2$	0.4333	-2.986 to 3.852	ns	0.9998
NH_4Y vs. NaY (S)	-2.233	-5.652 to 1.186	ns	0.3690
NH_4Y vs. NH_4Y (S)	0.8167	-2.602 to 4.236	ns	0.9887
NH_4Y-$CaCl_2$ vs. NaY(S)	-2.667	-6.086 to 0.7525	ns	0.1918
NH_4Y-$CaCl_2$ vs. NH_4Y (S)	0.3833	-3.036 to 3.802	ns	>0.9999
NaY(S) vs. NH_4Y (S)	3.050	-0.3691 to 6.469	ns	0.0990

All zeolite formulations applied either by the padded or sprayed (S) method for these swatches contain 0.5%pectin and 2% calcium chloride ($CaCl_2$) and either 5% NaY or 10% NH_4Y. Abbreviations are ns: not significant; combat gauze (CG), sodium Y zeolite (NaY), ammonium Y zeolite (NH_4Y), TACGauze (TGz), sprayed (S) and samples are padded otherwise. * p-value <0.05; ** p-value <0.01; *** p-value <0.001; **** p-value <0.0001, as determined by one-way ANOVA with Tukey's post hoc test.

Table S2. One-way ANOVA of R values shown in Figure 6b as determined by one-way ANOVA with Tukey's post hoc test.

Sample	Mean Diff.	95.00% CI of diff.	Summary	Adjusted p value
Control vs. TGz	3.533	-1.408 to 8.475	ns	0.2722
Control vs. CXTGz	8.417	3.475 to 13.36	***	0.0005
Control vs. BGz	4.617	-0.3251 to 9.558	ns	0.0762
Control vs. AgTGz	5.133	0.1916 to 10.08	*	0.0388
Control vs. CXTGz + Y	10.53	5.592 to 15.48	****	<0.0001
Control vs. BGz + Y	11.02	6.075 to 15.96	****	<0.0001
Control vs. AgTGz + Y	10.43	5.492 to 15.38	****	<0.0001
TGz vs. CXTGz	4.883	-0.05841 to 9.825	ns	0.054
TGz vs. BGz	1.083	-3.858 to 6.025	ns	0.9931
TGz vs. AgTGz	1.6	-3.342 to 6.542	ns	0.9426
TGz vs. CXTGz +Y	7	2.058 to 11.94	**	0.0031
TGz vs. BGz + Y	7.483	2.542 to 12.43	**	0.0016
TGz vs. AgTGz + Y	6.9	1.958 to 11.84	**	0.0035
CXTGz vs. BGz	-3.8	-8.742 to 1.142	ns	0.2039
CXTGz vs. AgTGz	-3.283	-8.225 to 1.658	ns	0.35
CXTGz vs. CXTGz + Y	2.117	-2.825 to 7.058	ns	0.8055
CXTGz vs. BGz + Y	2.6	-2.342 to 7.542	ns	0.616
CXTGz vs. AgTGz + Y	2.017	-2.925 to 6.958	ns	0.8389
BGz vs. AgTGz	0.5167	-4.425 to 5.458	ns	>0.9999
BGz vs. CXTGz + Y	5.917	0.9749 to 10.86	*	0.0135
BGz vs. BGz + Y	6.4	1.458 to 11.34	**	0.007
BGz vs. AgTGz + Y	5.817	0.8749 to 10.76	*	0.0155
AgTGz vs. CXTGz + Y	5.4	0.4583 to 10.34	*	0.0272
AgTGz vs. BGz + Y	5.883	0.9416 to 10.83	*	0.0141
AgTGz vs. AgTGz + Y	5.3	0.3583 to 10.24	*	0.0311
CXTGz + Y vs. BGz + Y	0.4833	-4.458 to 5.425	ns	>0.9999
CXTGz + Y vs. AgTGz + Y	-0.1	-5.042 to 4.842	ns	>0.9999
BGz + Y vs. AgTGz + Y	-0.5833	-5.525 to 4.358	ns	0.9999

Abbreviations are TACGauze (TGz), BIOGauze (BGz), silver TACGauze (AgTGz), citrate gauze (CXTGz), (Name)+Y = fabric with NH_4Y zeolite type, see Table 1 in manuscript in the "Materials and Methods" section for the details of the formularies. * p-value <0.05; ** p-value <0.01; *** p-value <0.001; **** p-value <0.0001, as determined by one-way ANOVA with Tukey's post hoc test, ns= non-significant.
